# Hypoxia and perfusion in breast cancer: simultaneous assessment using PET/MR imaging

**DOI:** 10.1007/s00330-020-07067-2

**Published:** 2020-07-28

**Authors:** Julia C. Carmona-Bozo, Roido Manavaki, Ramona Woitek, Turid Torheim, Gabrielle C. Baxter, Corradina Caracò, Elena Provenzano, Martin J. Graves, Tim D. Fryer, Andrew J. Patterson, Fiona J. Gilbert

**Affiliations:** 1 Department of Radiology, School of Clinical Medicine, University of Cambridge, Box 218, Cambridge Biomedical Campus, Cambridge, CB2 0QQ UK; 2 Department of Biomedical Imaging and Image-Guided Therapy, Medical University of Vienna, Währinger Gürtel 18-20, 1090 Vienna, Austria; 3 Cancer Research UK – Cambridge Institute, University of Cambridge, Li Ka Shing Centre, Robinson Way, Cambridge, CB2 0RE UK; 4 Cambridge Breast Unit, Cambridge University Hospitals NHS Foundation Trust, Box 97, Cambridge Biomedical Campus, Cambridge, CB2 0QQ UK; 5 MRIS Unit, Cambridge University Hospitals NHS Foundation Trust, Box 162, Cambridge Biomedical Campus, Cambridge, CB2 0QQ UK; 6 Wolfson Brain Imaging Centre, Department of Clinical Neurosciences, School of Clinical Medicine, University of Cambridge, Box 65, Cambridge Biomedical Campus, Cambridge, CB2 0QQ UK

**Keywords:** PET/MRI, Hypoxia, Perfusion, Breast cancer

## Abstract

**Objectives:**

Hypoxia is associated with poor prognosis and treatment resistance in breast cancer. However, the temporally variant nature of hypoxia can complicate interpretation of imaging findings. We explored the relationship between hypoxia and vascular function in breast tumours through combined ^18^F-fluoromisonidazole (^18^ F-FMISO) PET/MRI, with simultaneous assessment circumventing the effect of temporal variation in hypoxia and perfusion.

**Methods:**

Women with histologically confirmed, primary breast cancer underwent a simultaneous ^18^F-FMISO-PET/MR examination. Tumour hypoxia was assessed using influx rate constant *K*_i_ and hypoxic fractions (%HF), while parameters of vascular function (*K*^trans^, *k*_ep_, *v*_e_, *v*_p_) and cellularity (ADC) were derived from dynamic contrast-enhanced (DCE) and diffusion-weighted (DW)-MRI, respectively. Additional correlates included histological subtype, grade and size. Relationships between imaging variables were assessed using Pearson correlation (*r*).

**Results:**

Twenty-nine women with 32 lesions were assessed. Hypoxic fractions > 1% were observed in 6/32 (19%) cancers, while 18/32 (56%) tumours showed a %HF of zero. The presence of hypoxia in lesions was independent of histological subtype or grade. Mean tumour *K*^trans^ correlated negatively with *K*_i_ (*r* = − 0.38, *p* = 0.04) and %HF (*r* = − 0.33, *p* = 0.04), though parametric maps exhibited intratumoural heterogeneity with hypoxic regions colocalising with both hypo- and hyperperfused areas. No correlation was observed between ADC and DCE-MRI or PET parameters. %HF correlated positively with lesion size (*r* = 0.63, *p* = 0.001).

**Conclusion:**

Hypoxia measured by ^18^F-FMISO-PET correlated negatively with *K*^trans^ from DCE-MRI, supporting the hypothesis of perfusion-driven hypoxia in breast cancer. Intratumoural hypoxia-perfusion relationships were heterogeneous, suggesting that combined assessment may be needed for disease characterisation, which could be achieved using simultaneous multimodality imaging.

**Key Points:**

• *At the tumour level, hypoxia measured by *^*18*^*F-FMISO-PET was negatively correlated with perfusion measured by DCE-MRI, which supports the hypothesis of perfusion-driven hypoxia in breast cancer.*

• *No associations were observed between 18F-FMISO-PET parameters and tumour histology or grade, but tumour hypoxic fractions increased with lesion size.*

• *Intratumoural hypoxia-perfusion relationships were heterogeneous, suggesting that the combined hypoxia-perfusion status of tumours may need to be considered for disease characterisation, which can be achieved via simultaneous multimodality imaging as reported here.*

**Electronic supplementary material:**

The online version of this article (10.1007/s00330-020-07067-2) contains supplementary material, which is available to authorized users.

## Introduction

Hypoxia is a common characteristic of the tumour microenvironment and arises due to avid metabolism and poor perfusion as a result of the structurally and functionally aberrant microcirculation found in tumours [1]. In breast cancer, the presence of hypoxia has been confirmed with pO_2_ histography and occurs irrespective of histological type, molecular subtype, grade or patient characteristics [2, 3]. In vitro studies have shown that hypoxia promotes a dedifferentiated phenotype in ductal carcinoma in situ [4] and downregulates the expression and function of oestrogen receptor-α (ERα) [5]. Several clinical and preclinical studies in breast cancer have demonstrated that overexpression of hypoxia-related proteins is associated with an aggressive phenotype, poor prognosis and resistance to treatment [6–8].

Although tumour hypoxia can be broadly categorised as diffusion or perfusion limited, it is generally accepted that the tumour microenvironment is a highly dynamic entity, exhibiting temporally varying perfusion patterns and heterogeneous oxygen-tension gradients [9]. Experimental evidence suggests that oxygen levels continually fluctuate owing to transient changes in perfusion [10]. These changing perfusion and oxygenation levels induce a variety of gene expression profiles resulting in a unique micromilieu that is pivotal for tumour growth and metastatic dissemination [11]. Given the temporal variation in oxygenation and perfusion within tumours, sequential multimodal imaging investigations may not always be effective in assessing the association between these parameters, as similar tumour status cannot be guaranteed between imaging sessions. Simultaneous assessment of the hypoxia and perfusion in tumours can mitigate confounders associated with the dynamic character of these processes, and thus allow additional pathophysiological characterisation of breast cancer.

Imaging methods, including positron emission tomography (PET) and magnetic resonance imaging (MRI), have been used for the non-invasive assessment of the tumour microenvironment. Dynamic contrast-enhanced (DCE) MRI has shown utility in characterising tumour perfusion and vascular permeability in clinical studies [12], while diffusion-weighted imaging (DWI) can provide surrogate measures of tumour cellular density [13]. PET with ^18^F-labelled nitroimidazoles can provide specific measures of intracellular hypoxia [14]. In breast cancer, ^18^F-fluoromisonidazole (^18^F-FMISO) has been used for the evaluation of response to anti-angiogenic and HER2-targetted treatment [15, 16] and shown potential utility as a predictor of response to primary endocrine therapy [17, 18]. Additionally, high ^18^F-FMISO uptake at baseline has been associated with shorter disease-free survival [18] and disease-specific death [19].

Despite the intrinsic link between tumour hypoxia and perfusion, multimodal imaging approaches to characterise this aspect of cancer pathophysiology have been limited in the clinical setting [16, 19–24]. To effectively assess relationships between temporally varying microenvironment parameters, combined PET/MR imaging presents an attractive option as it permits examination of the tumour under the same physiologic conditions, while also conferring methodological advantages in the spatial registration of data from the two modalities.

The primary objective of this study was to examine the association between hypoxia and vascular function in patients with treatment-naïve breast cancer using simultaneous ^18^F-FMISO-PET/MRI. To our knowledge, this is the first such study in breast cancer.

## Materials and methods

### Study participants

Women aged > 18 years with histologically confirmed primary breast cancer and a tumour diameter > 10 mm on mammography and/or ultrasound were eligible for the study (February 2017 to November 2018). Pregnancy, lactation, previous surgery or radiotherapy for cancer or benign breast disease, inadequate renal function and contraindications to MRI were exclusion criteria for the study. The research was approved by a National Research Ethics Committee (14/EE/0145). All study participants provided written informed consent before PET/MRI examination.

### PET/MRI acquisition

Participants underwent a 60-min simultaneous PET/MR scan of the breasts in the prone position on a SIGNA PET/MR scanner (GE Healthcare), using a 16-channel bilateral breast array (RAPID Biomedical) 120 min (median [range], 120.2 [119.8–127.5] min) after injection of 306 ± 14 MBq ^18^F-FMISO. The uptake period post injection (p.i.) was used to enhance hypoxic-to-normoxic tissue contrast and allow the free ^18^F-FMISO concentrations in tissue and blood to reach equilibrium [25, 26], a requirement for influx rate constant (*K*_i_) determination by Patlak analysis [27].

*PET*: Emission data from 120 to 180 min p.i. (12 × 5-min frames) were reconstructed using time-of-flight ordered-subsets expectation-maximisation (TOF-OSEM) with 4 iterations and 28 subsets (Supplemental Methods I). Plasma radioactivity concentration from two venous blood samples, acquired immediately before and after PET/MR acquisition, was used to scale a ^18^F-FMISO population-based arterial input function (AIF) derived from existing data, permitting calculation of *K*_i_ [28] (Supplemental Methods II; Supplemental Fig. 1; [29–31]).

*MRI*: The MRI protocol involved a 2-point Dixon sequence for PET attenuation correction, T_1_- and T_2_-weighted images, DWI, and a DCE series. Sequences were also acquired to measure B_1_^+^ transmission-field non-uniformity, using a Bloch-Siegert method, and baseline T_1_ (T_10_) as required for the pharmacokinetic analysis of DCE-MRI data [32]. DCE-MRI acquisition involved five pre-contrast images, followed by 43 phases after intravenous bolus injection of 0.1 mmol/kg of Gadovist (Bayer Healthcare). MRI sequence details are given in Supplemental Table 1.

### Image analysis

Tumour regions were manually delineated in OsiriX, version 8.0.2 (Pixmeo SARL), by three radiologists in consensus (1, 3 and > 20 years of experience in breast MRI). Regions were drawn on the peak-enhancing volumes of the DCE-MRI series on all contiguous axial sections encompassing the invasive part of the tumour and including multifocal/multicentric disease (Supplemental Methods III). Synchronous bilateral cancers were regarded as independent lesions [33].

*DCE-MRI*: Pharmacokinetic analysis of the DCE-MRI series was performed in MIStar, version 3.2.63 (Apollo Medical Imaging), using the extended Tofts’ model [34] to calculate contrast influx rate constant, *K*^trans^; efflux-rate constant, *k*_ep_; extravascular-extracellular volume fraction, *v*_e_; and plasma volume fraction, *v*_p_ (Supplemental Methods III).

*DWI*: Calculation of apparent diffusion coefficient (ADC) maps was performed in OsiriX, using *b* values of 0 and 900 s/mm^2^. Mean lesion ADC was calculated by manually outlining whole tumour regions on the *b* = 900 s/mm^2^ image (Supplemental Methods III; [35] ).

*PET*: Image frames from 150 to 180 min p.i. were averaged, rigidly registered to the peak-enhancing phase of the DCE-MRI series and subsequently employed for the determination of ^18^F-FMISO uptake as mean and maximum standardised uptake values normalised by body weight (SUV_mean_, SUV_max_) and maximum tumour-to-plasma (*T*_max_/*P*) and tumour-to-muscle (*T*_max_/*M*) ratios within the regions defined on the DCE-MRI. The influx rate of ^18^F-FMISO into the trapped (hypoxic) tissue compartment (*K*_i_) was determined by Patlak-plot analysis, utilising all frames in the registered ^18^F-FMISO series and the scaled population-based AIF. Hypoxic fractions (%HF) in tumour regions were calculated as the percentage of voxels with *K*_i_ values > 2 × standard deviations (SD) of the mean *K*_i_ of normoxic muscle (Supplemental Methods III).

### Histology

Histopathological information including tumour histological subtype, grade, oestrogen receptor (ER), progesterone receptor (PR) and human epidermal growth factor receptor-2 (HER2) status was obtained from core biopsies or surgical tumour specimens. Cancers with positive ER or PR expression were classified as hormone-receptor (HR) positive.

### Statistics

Statistical analysis was performed in IBM SPSS Statistics for MacOS, v25.0 (IBM Corp.) or Matlab 2016b. Continuous data were assessed for normality using the Anderson-Darling test. Correlations between continuous variables were assessed using the Pearson correlation coefficient (*r*). *t* tests were used for comparison between means of two groups, and ANOVA when more than two groups were compared. Where data were not normally distributed, or normality could not be assessed, Mann-Whitney *U* and Mood’s median or Kruskal-Wallis *H* tests were employed for comparisons between two or more groups, respectively. *p* values < 0.05 were considered statistically significant.

## Results

A total of 32 women were enrolled into the study. Two participants withdrew before the PET/MR examination. PET/MRI data and DCE-MRI data from two participants were excluded owing to inadequate acquisition of DCE-MRI and poor pharmacokinetic-model fitting respectively. In total, data from 29 participants with 32 biopsy-confirmed primary breast cancers were analysed. ADC calculations included data from 18 patients (19 lesions), who successfully completed the DWI examination.

Two thirds of the lesions (21/32; 66%) were invasive ductal cancers (IDC). The majority of cancers (29/32; 91%) were either grade 2 or 3. HR-positive expression was noted for 31/32 (97%) lesions, with 24/32 (77%) cancers being HER2-negative. Tumour characteristics are summarised in Table 1. Additional clinical information is provided in Supplemental Table 2.

**Table 1:** Clinical characteristics for the patient population (*n* = 29)

Characteristic	*n* (%)
Age at diagnosis (years)^a^	57 [37–78]
Lesions	32
Pathological size (mm)^a, b^	26 [10–142]
Lesion longest diameter on MRI	
≤ 20 mm	10 (31)
> 20 mm	22 (69)
Histopathological subtype	
Ductal (IDC)	21 (66)
Lobular (ILC)	6 (19)
Mucinous (IMC)	2 (6)
Mixed^c^	3 (9)
Histological grade^d^	
1	3 (9)
2	16 (50)
3	13 (41)
Hormone-receptor status^e^	
Positive (ER or PR)	31 (97)
Negative	1 (3)
HER2 status^f^	
Positive	7 (22)
Negative	25 (78)

*ER* oestrogen receptor, *PR* progesterone receptor, *HER2* human epidermal growth factor receptor 2

^a^Data presented as median [range]

^b^Pathological size measured on tumour specimens from patients undergoing primary surgery (*n* = 21)

^c^Invasive carcinomas with presence of both lobular and ductal components on histology

^d^Nottingham combined histologic grade

^e^Tumours classified as ER or PR-positive, if > 10% of the cells demonstrated nuclear staining by immunohistochemistry

^f^Tumours classified as HER2-positive, if they scored 3+ on immunohistochemistry or if they carried gene amplification as detected by fluorescence in situ hybridisation (FISH)

### Relationship between ^18^F-FMISO-PET and DCE-MRI parameters

**Fig. 1:**
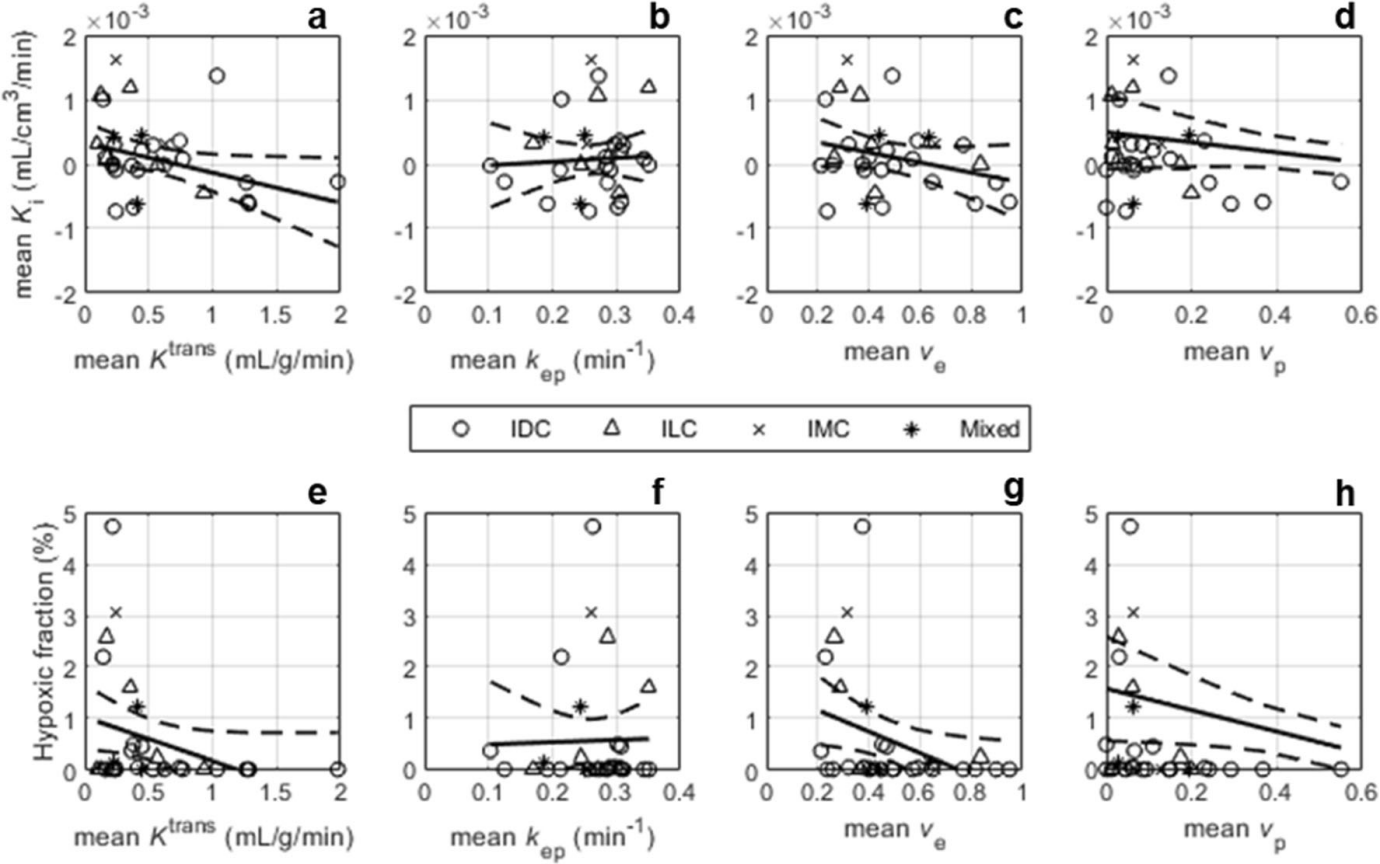
^18^F-FMISO-PET *K*_i_ and hypoxic fraction (%) vs. the following DCE-MRI parameters: (**a**, **e**) contrast influx rate, *K*^trans^ (mL/g/min); (**b**, **f**) contrast efflux rate, *k*_ep_ (min^−1^); (**c**, **g**) fractional volume of extravascular-extracellular space, *v*_e_; (**d**, **h**) plasma fractional volume, *v*_p_. IDC, invasive ductal carcinoma; ILC, invasive lobular carcinoma; IMC, invasive mucinous carcinoma; Mixed, carcinoma of mixed ductal and lobular type

Scatter plots indicating the relationships between DCE-MRI parameters and *K*_i_ or %HF are illustrated in Fig. 1. An inverse relationship was observed between mean lesion *K*_i_ and *K*^trans^, *v*_e_ and *v*_p_ (Fig. 1(a–d); Supplemental Fig. 2 [36]), which was statistically significant for *K*_i_ vs. *K*^trans^ (*r* = − 0.38, *p* = 0.04), but not for *K*_i_ vs. *v*_e_ (*r* = − 0.30, *p* = 0.10) or *v*_p_ (*r* = − 0.28, *p* = 0.12). Associations between %HF and DCE-MRI parameters followed similar trends, also indicating a decrease in hypoxia with increasing *K*^trans^, *v*_e_ and *v*_p_ (Fig. 1(e–h)). Statistically significant correlations were observed between %HF and both *K*^trans^ (*r* = − 0.33, *p* = 0.04) and *v*_e_ (*r* = − 0.38, *p* = 0.03). No correlation was observed between *k*_ep_ and either *K*_i_ (*r* = 0.08, *p* = 0.65) or %HF (*r* = 0.02, *p* = 0.90).

**Fig. 2:**
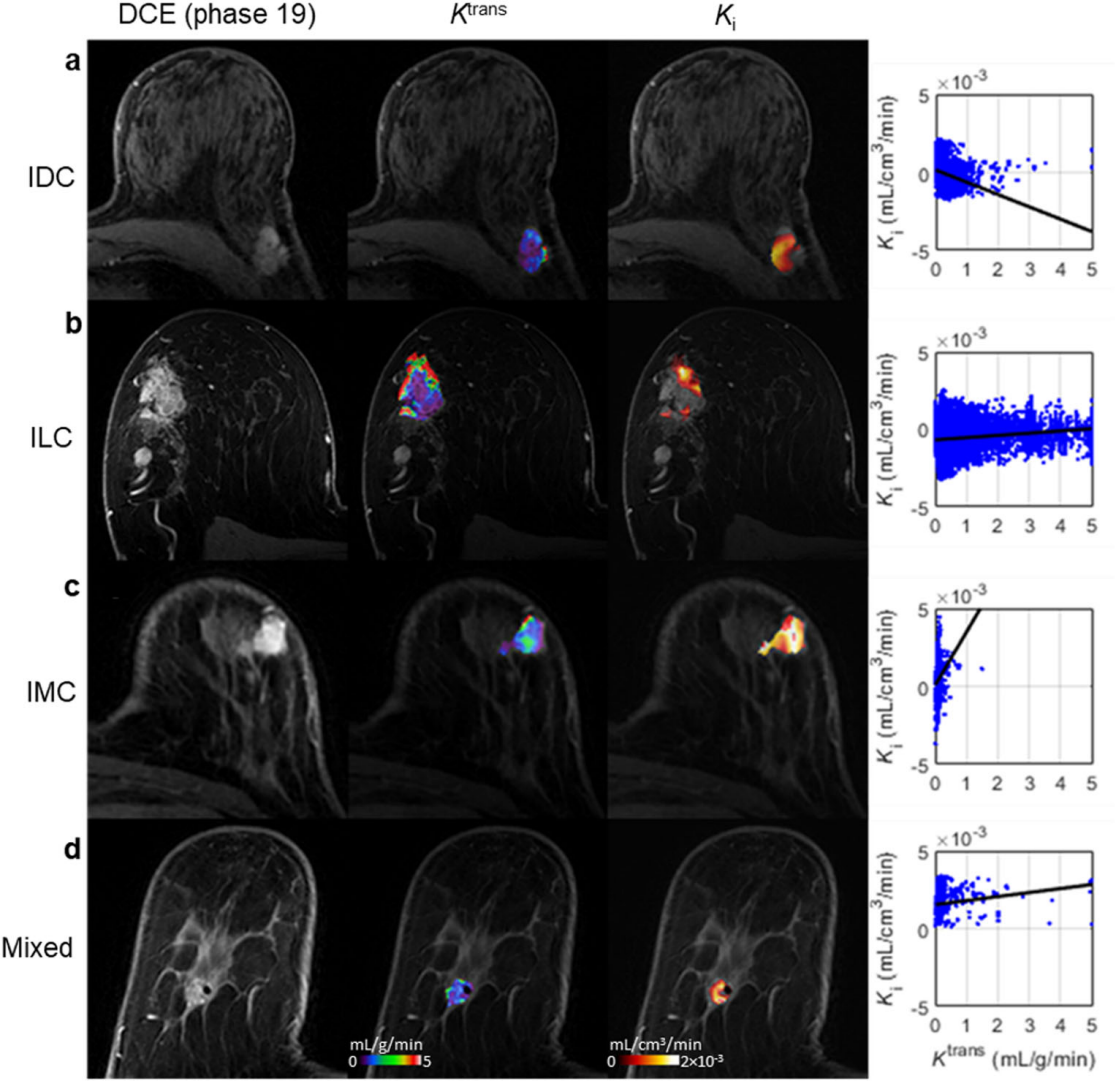
Axial images of four representative patients with: **a** invasive ductal carcinoma (IDC); **b** invasive lobular carcinoma (ILC); **c** invasive mucinous carcinoma (IMC); and **d** carcinoma of mixed ductal and lobular type (Mixed). (*Left-to-right*) DCE-MRI image at peak enhancement; *K*^trans^ map representing tumour perfusion for the lesion ROI overlaid on the peak-enhancing DCE-MRI image; *K*_i_ map representing tumour hypoxia for the lesion ROI overlaid on the peak-enhancing DCE-MRI image; scatter plot and regression line of *K*_i_ vs. *K*^trans^ voxel values within the tumour. *K*^trans^, contrast influx rate (mL/g/min); *K*_i_, ^18^F-FMISO influx rate (mL/cm^3^/min)

Figure 2 presents axial slices through *K*_i_ and *K*^trans^ parametric maps of four tumours of different histological subtypes, indicating heterogeneous spatial relationships between hypoxia and perfusion; other DCE-MRI parametric images are given in Supplemental Fig. 3.

### ^18^F-FMISO-PET and DCE-MRI parameters vs. tumour histology and grade

**Fig. 3:**
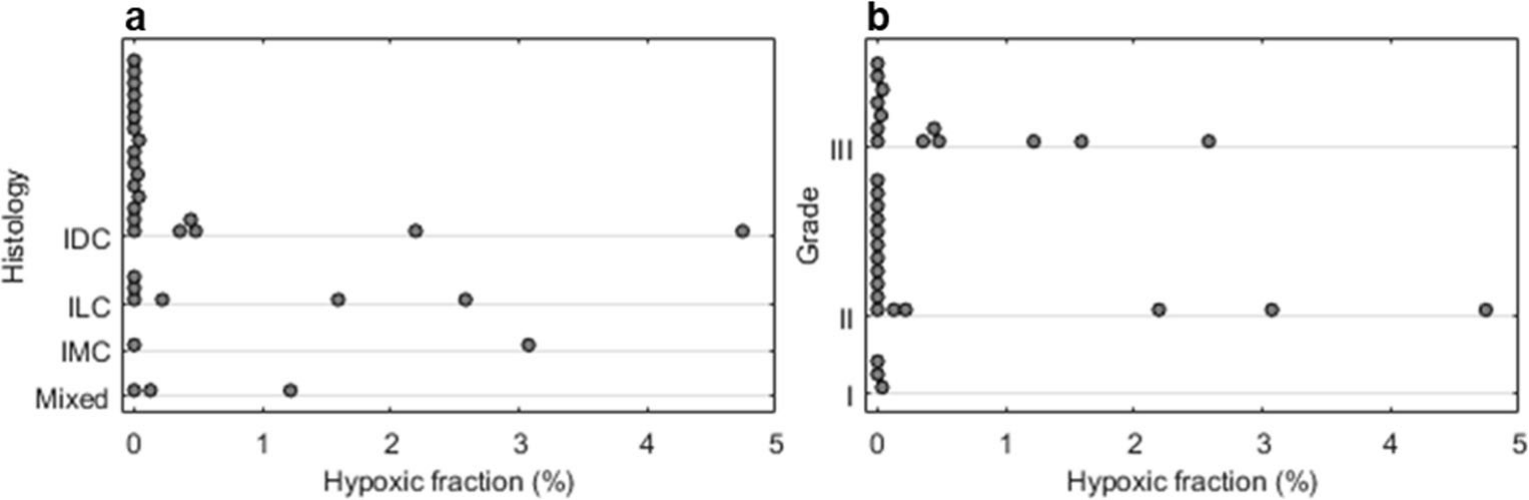
Dot plots of hypoxic fraction (%) by (**a**) histological type and (**b**) nuclear grade

Hypoxic fractions > 1% were observed in 6/32 (19%) cancers with an additional 8/32 (25%) lesions displaying hypoxic fractions greater than zero but less than 1%; the remaining 18/32 (56%) tumours had a %HF of zero. Dot plots of %HF vs. tumour histological subtype and grade are presented in Fig. 3. *K*_i_, %HF and ^18^F-FMISO uptake parameters showed no significant difference between different histological subtypes or grades (Tables 2 and 3). Similarly, no significant differences were observed between histological groups or grades for the DCE-derived parameters (Tables 2 and 3), except for the efflux rate constant *k*_ep_, which displayed a statistically significant difference among grade 2 and 3 cancers (median [range], 0.25 [0.13–0.34] vs. 0.30 [0.10–0.35] min^−1^; *p* = 0.01). Furthermore, analysis of hypoxia and *K*^trans^ values in the most vascularised area of the tumour (hotspot on DCE-MRI) yielded no significant differences among different subtypes or grades (Supplemental Tables 3 and 4).

**Table 2:** MRI and ^18^F-FMISO-PET parameters with respect to tumour histology. Data are presented as median [range] or mean ± standard deviation (SD) as appropriate

Parameter	Histology				*p* value
	IDC	ILC	Mixed	IMC	
Lesions (*n* = 31)	20	6	3	2	
*K*^trans^	0.43 [0.14–1.97]	0.26 [0.10–0.94]	0.41 [0.23–0.45]	0.44 [0.25–0.64]	0.77^a^
*k*_ep_	0.26 [0.10–0.35]	0.28 [0.17–0.35]	0.25 [0.19–0.25]	0.26 [0.25–0.26]	0.14^a^
*v*_e_	0.46 [0.21–0.95]	0.39 [0.26–0.84]	0.44 [0.39–0.64]	0.49 [0.31–0.66]	0.30^a^
*v*_p_	0.08 [0–0.55]	0.05 [0.01–0.2]	0.06 [0.03–0.19]	0.09 [0.06–0.13]	0.77^a^
Lesions (*n* = 19)	14	3	1	1	
ADC (× 10^−3^)	0.90 [0.42–1.55]	1.05 [0.84–1.28]	1.02 [−]	2.46 [−]	0.51^b^
Lesions (*n* = 32)	21	6	3	2	
*K*_i_ (× 10^−3^)	0.00 ± 0.52	0.37 ± 0.65	0.08 ± 0.61	0.97 ± 0.91	0.26^c^
%HF	0 [0–4.74]	0.10 [0–2.58]	0.13 [0–1.22]	1.54 [0–3.07]	0.63^a^
SUV_max_	1.53 ± 0.41	1.77 ± 0.16	1.60 ± 0.21	1.25 ± 0.12	0.31^c^
SUV_mean_	1.14 ± 0.26	1.27 ± 0.18	1.17 ± 0.16	1.07 ± 0.15	0.65^c^
*T*_max_/*M*	1.02 ± 0.24	1.30 ± 0.29	1.09 ± 0.22	0.95 ± 0.02	0.12^c^
*T*_max_/*P*	0.87 ± 0.22	0.83 ± 0.33	0.87 ± 0.09	0.84 ± 0.09	0.99^c^

*IDC* invasive ductal carcinoma, *ILC* invasive lobular carcinoma, *Mixed* invasive carcinoma with presence of lobular and ductal components, *IMC* invasive mucinous carcinoma, *K*^*trans*^ contrast influx rate (mL/g/min), *k*_*ep*_ contrast efflux rate (min^−1^), *v*_*e*_ fractional volume of extravascular-extracellular space, *v*_*p*_ plasma fractional volume, *ADC* apparent diffusion coefficient (mm^2^/s), *K*_*i*_^18^F-FMISO influx rate (mL/cm^3^/min), *%HF* percentage hypoxic fraction, *SUV* standardised uptake value (g/mL), *T*_*max*_*/M* maximum tumour-to-muscle ratio, *T*_*max*_*/P* maximum tumour-to-plasma ratio

^a^Mood’s median test

^b^Mann-Whitney *U* test for malignancies of type IDC and ILC only (mixed and IMC lesions were not included in the comparison)

^c^One-way analysis of variance (ANOVA)

**Table 3:** MRI and ^18^F-FMISO-PET parameters with respect to nuclear grade. Data are presented as median [range] or mean ± standard deviation (SD) as appropriate

Parameter	Grade			*p* value
	1	2	3	
Lesions (*n* = 31)	3	15	13	
*K*^trans^	0.41 [0.24–0.54]	0.24 [0.10–1.98]	0.45 [0.17–1.27]	0.29^a^
*k*_ep_	0.29 [0.26-0.31]	0.25^*c^ [0.13–0.34]	0.30^*d^ [0.10–0.35]	0.009^**a^
*v*_e_	0.38 [0.24–0.77]	0.45 [0.23–0.84]	0.43 [0.21–0.95]	0.65^a^
*v*_p_	0.06 [0.05-0.08]	0.06 [0.00–0.55]	0.09 [0.00–0.37]	0.46^a^
Lesions (*n* = 19)	1	9	9	
ADC (× 10^−3^)	1.08 [−]	1.05 [0.42–2.46]	0.84 [0.70–1.28]	0.34^b^
Lesions (*n* = 32)	3	16	13	
*K*_i_ (× 10^−3^)	− 0.18 ± 0.52	0.25 ± 0.58	0.06 ± 0.65	0.47^c^
%HF	0 [0-0.04]	0 [0–4.74]	0.04 [0–2.6]	0.35^a^
SUV_max_	1.28 ± 0.29	1.55 ± 0.29	1.66 ± 0.46	0.28^c^
SUV_mean_	0.98 ± 0.09	1.18 ± 0.19	1.18 ± 0.29	0.37^c^
*T*_max_/*M*	0.96 ± 0.02	1.04 ± 0.17	1.56 ± 0.36	0.36^c^
*T*_max_/*P*	0.78 ± 0.08	0.81 ± 0.20	0.85 ± 0.25	0.21^c^

*K*^*trans*^ contrast influx rate (mL/g/min), *k*_*ep*_ contrast efflux rate (min^−1^), *v*_*e*_ fractional volume of extravascular-extracellular space, *v*_*p*_ plasma fractional volume, *ADC* apparent diffusion coefficient (mm^2^/s), *K*_*i*_^18^F-FMISO influx rate (mL/cm^3^/min), *%HF* percentage hypoxic fraction (%), *SUV* standardised uptake value (g/mL), *T*_*max*_*/M* maximum tumour-to-muscle ratio, *T*_*max*_*/P* maximum tumour-to-plasma ratio

^*^p < 0.05; ^**^p < 0.01

^a^Kruskal-Wallis *H*

^b^Mann-Whitney *U* test for grade 1 and 2 cancers only (grade 1 lesions were not included in the comparison)

^c^One-way analysis of variance (ANOVA)

^d^Significant difference between grade 2 and 3 cancers (*p* = 0.01). Pairwise multiple comparison analysis utilised the Dwass-Steel-Critchlow-Fligner method

### Effect of tumour size on ^18^F-FMISO-PET and DCE-MRI parameters

Table 4 presents correlations between imaging indices and tumour size as measured by longest diameter on MRI or pathological size. No or weak negative correlations were observed between tumour size and DCE-MRI parameters. Conversely, ^18^F-FMISO-PET parameters correlated positively with size; %HF significantly correlated with pathological size (*r* = 0.63, *p* = 0.001), while ^18^F-FMISO-PET uptake metrics displayed associations of moderate strength with longest diameter on MRI.

**Table 4:** Pearson correlation coefficient *r* (*p* value) between tumour size, MRI and ^18^F-FMISO-PET parameters

Parameter	Tumour size (mm)	
	Longest diameter on MRI	Pathological size^a^
Lesions (*n*)	31	21
*K*^trans^	− 0.15 (0.42)	− 0.16 (0.48)
*k*_ep_	− 0.04 (0.84)	− 0.15 (0.48)
*v*_e_	− 0.04 (0.83)	− 0.27 (0.22)
*v*_p_	− 0.13 (0.50)	− 0.09 (0.70)
Lesions (*n*)	19	11
ADC (× 10^−3^)	0.06 (0.80)	0.56 (0.07)
Lesions (*n*)	32	21
*K*_i_ (×10^−3^)	0.15 (0.29)	0.21 (0.48)
HF (%)	0.26 (0.16)	0.63 (0.001^**^)
SUV_max_	0.48 (0.02^*^)	0.26 (0.24)
SUV_mean_	0.42 (0.006^**^)	0.39 (0.07)
*T*_max_/*M*	0.45 (0.01^*^)	0.32 (0.14)
*T*_max_/*P*	0.43 (0.02^*^)	0.49 (0.02^*^)

*K*^*trans*^ contrast influx rate (mL/g/min), *k*_*ep*_ contrast efflux rate (min^−1^), *v*_*e*_ fractional volume of extravascular-extracellular space, *v*_*p*_ plasma fractional volume, *ADC* apparent diffusion coefficient (mm^2^/s), *K*_*i*_^18^F-FMISO influx rate (mL/cm^3^/min), *%HF* percentage hypoxic fraction, *SUV* standardised uptake value (g/mL), *T*_*max*_*/M* maximum tumour-to-muscle ratio, *T*_*max*_*/P* maximum tumour-to-plasma ratio

^*^p < 0.05; ^**^p < 0.01

^a^Pathological size as measured on tumour specimens from patients undergoing primary surgery (*n* = 21)

### ADC vs. ^18^F-FMISO-PET and DCE-MRI parameters

**Fig. 4:**
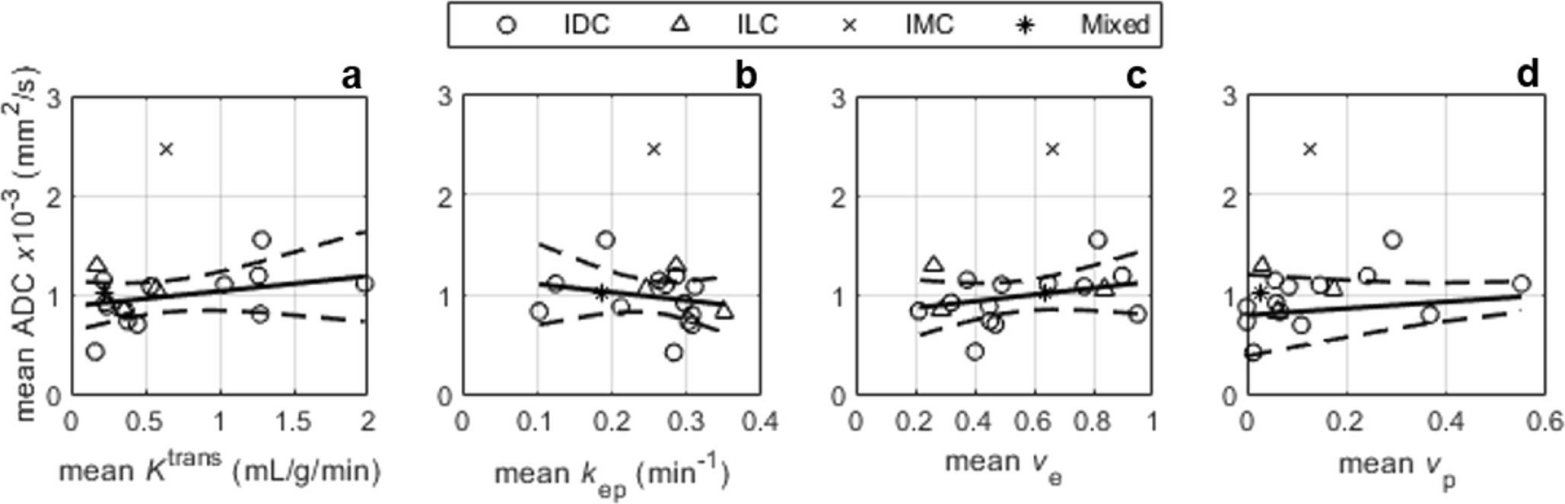
Apparent diffusion coefficient (ADC) vs. DCE-MRI parameters: **a** contrast influx rate, *K*^trans^; **b** contrast efflux rate, *k*_ep_; **c** fractional volume of extravascular-extracellular space, *v*_e_; **d** plasma fractional volume, *v*_p_. IDC, invasive ductal carcinoma; ILC, invasive lobular carcinoma; IMC, invasive mucinous carcinoma; Mixed, carcinoma of mixed ductal and lobular type

**Fig. 5:**
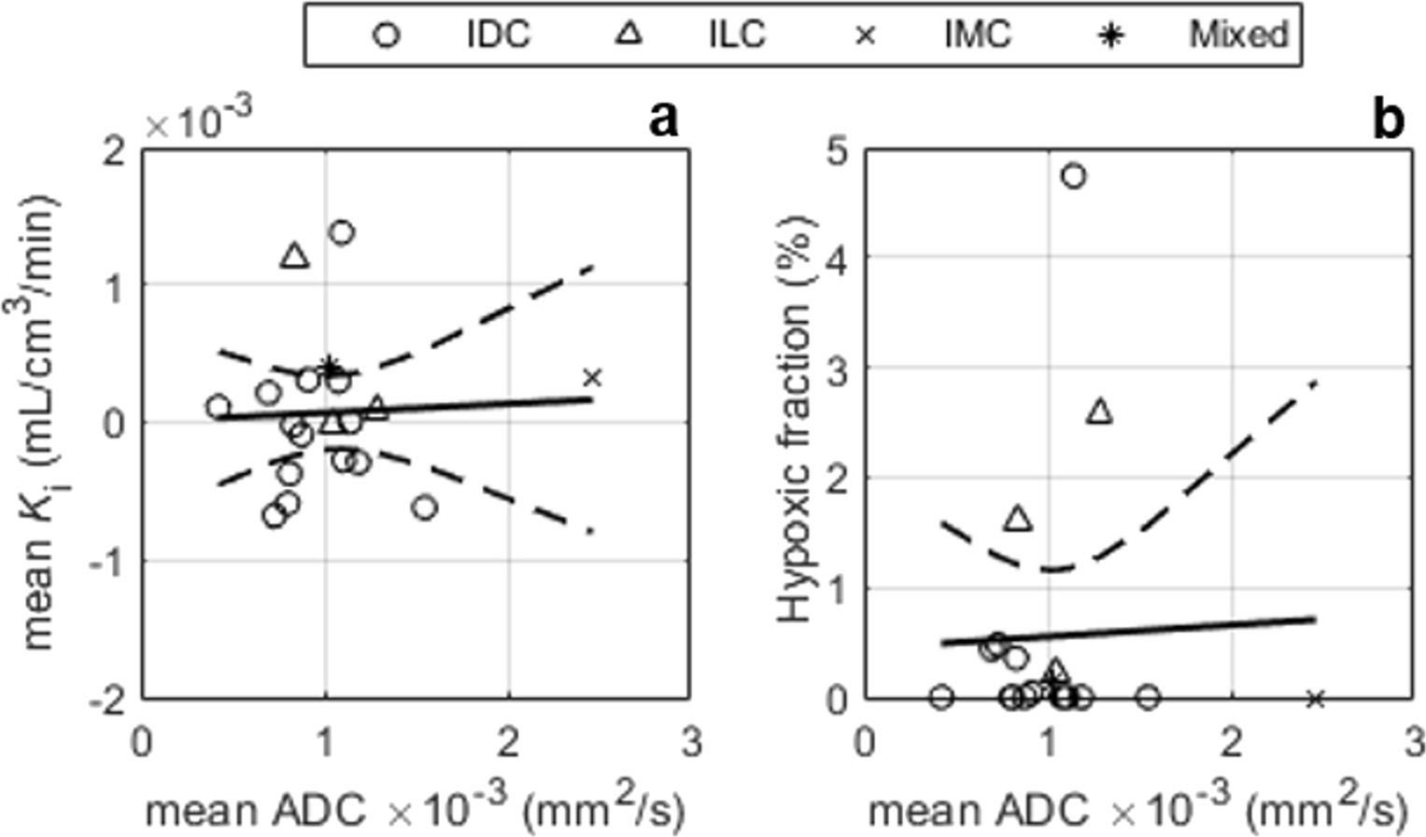
^18^F-FMISO-PET parameters vs. apparent diffusion coefficient (ADC): (**a**) influx rate *K*_i_ and (**b**) hypoxic fraction (%). IDC, invasive ductal carcinoma; ILC, invasive lobular carcinoma; IMC, invasive mucinous carcinoma; Mixed, carcinoma of mixed ductal and lobular type

**Fig. 6:**
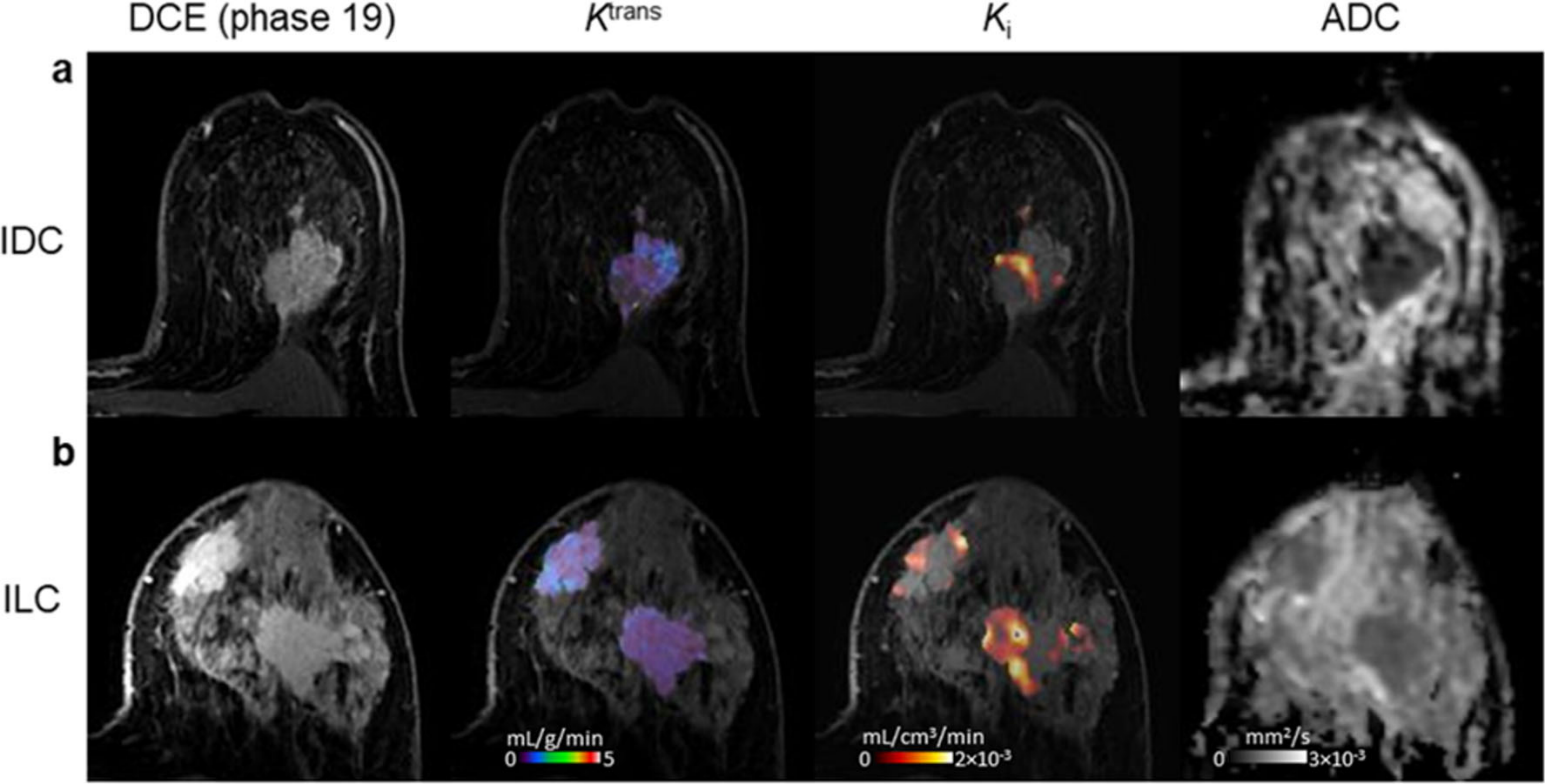
Axial images of two patients with: **a** invasive ductal carcinoma (IDC); **b** invasive lobular carcinoma (ILC). (*Left-to-right*) DCE-MRI image at peak enhancement; *K*^trans^ map representing tumour perfusion for the lesion ROI overlaid on the peak-enhancing DCE-MRI image; *K*_i_ map representing tumour hypoxia for the lesion ROI overlaid on the DCE-MRI image at peak enhancement; ADC map. *K*^trans^, contrast influx rate (mL/g/min); *K*_i_, ^18^F-FMISO influx rate (mL/cm^3^/min); ADC, apparent diffusion coefficient (mm^2^/s)

Positive correlations were observed between ADC and DCE-MRI indices (*K*^trans^: *r* = 0.24, *p* = 0.34; *v*_e_: *r* = 0.29, *p* = 0.25; *v*_p_: *r* = 0.20, *p* = 0.43), except for *k*_ep_ which correlated negatively with ADC (*r* = − 0.15, *p* = 0.56; Fig. 4), none of which were statistically significant. No correlations were observed between ADC and *K*_i_ or %HF (*K*_i_: *r* = 0.05, *p* = 0.84; %HF *r* = 0.04, *p* = 0.88; Fig. 5). Representative ADC maps are given in Fig. 6.

## Discussion

This study explored the relationship between tumour hypoxia and vascular function in breast cancer using combined ^18^F-FMISO-PET/MRI. Hypoxic fractions and *K*_i_ measured on ^18^F-FMISO-PET showed inverse relationships with the DCE-MRI perfusion parameter *K*^trans^, consistent with the generally accepted view that tumour hypoxia is a consequence of inadequate oxygen supply to the tumour [1]. Previous clinical studies in cervical and head-and-neck carcinomas have demonstrated significant negative correlations between contrast enhancement or pharmacokinetic parameters from DCE-MRI and polarographic pO_2_ measurements or pimonidazole immunohistochemistry [37, 38]. These findings are consistent with our results in breast cancer.

However, PET and DCE-MRI parametric images exhibited largely heterogeneous intratumoural patterns with hypoxic islands on *K*_i_ maps often colocalising with areas of increased *K*^trans^. This spatially discrepant relationship between hypoxia and perfusion has been previously documented, with the co-existence of hypoxic and hyperperfused tumour sub-volumes [39]. Various biological mechanisms, including hypoxia-induced angiogenesis, interstitial fluid pressure, a fluctuating haemodynamic response, increased oxygen diffusion distances from the microvasculature and the presence of longitudinal oxygen gradients across tumour vessels, have all been proposed to explain the occurrence of hypoxia in highly perfused areas [40, 41]. Thus, although the general trend of our results would support the widely accepted view that hypoxia develops in hypoperfused breast tumours, the diverse relationships observed in individual tumour sub-volumes indicate heterogeneity in hypoxia-perfusion patterns and reflect the variety of pathophysiological mechanisms occurring in cancers.

The weak relationship between PET hypoxia parameters with *k*_ep_ suggests that the degree of tumour hypoxia is more strongly influenced by vascular flow rather than vessel permeability. Li et al [42] have previously suggested that *k*_ep_ is a much more sensitive measure of vessel permeability than *K*^trans^, as the latter represents a combined measure of blood flow, vessel permeability and capillary-surface area. Our findings broadly agree with previous research in cervical and head-and-neck carcinomas, which illustrated weaker correlations between hypoxia and permeability-surface-area product than between hypoxia and blood flow [37, 43]. The relationship between *K*^trans^ and regional hypoxia observed in our study suggests this is due to fluctuations in tumour vascular flow rather than capillary permeability.

No or weak positive correlations were found between static ^18^F-FMISO parameters (SUV_mean_, SUV_max_, *T*_max_/*M*, *T*_max_/*P*) and DCE-MRI metrics. In contrast, in human head-and-neck cancer, where hypoxia is often marked, ^18^F-FMISO SUV measurements were negatively correlated with both *K*^trans^ and *k*_ep_ [20]. A plausible explanation for this disparity is the higher level of hypoxia typically encountered in head-and-neck cancer, which will lead to uptake values being more dominated by hypoxia-specific ^18^F-FMISO trapping rather than non-specific tracer accumulation. Due to the higher contribution of non-specific ^18^F-FMISO accumulation at low hypoxia levels [44], the use of uptake values in cancers without marked hypoxia may not accurately reveal relationships between hypoxia and perfusion.

No significant correlation was observed between PET hypoxia parameters and tumour grade or subtype. Our sample size of non-IDC cases was small for evaluating the impact of histology on tumour hypoxic status, but the presence of non-zero hypoxic fractions was observed in all histological subtypes studied. Hypoxic fractions and higher *K*_i_ were noted in both grade 2 and 3 tumours, and less so in grade 1 cancers. These findings are concordant with previously reported small differences in hypoxia between low- and high-grade breast malignancy [2].

Correlations between DCE-MRI functional parameters and pathological size or MR tumour diameter yielded moderate negative relationships and conversely positive associations between ^18^F-FMISO-PET hypoxia parameters and size. The size-related hypoxia changes could be ascribed to diffusion-limited hypoxia, concomitant perfusion decreases or increased interstitial fluid pressure [45].

ADC has been shown to inversely correlate with cellular density [46], and therefore, a reduction in ADC should theoretically be accompanied by an increase in tumour hypoxia. Our findings indicated no association between ADC and PET hypoxia parameters. This result could be explained by the molecular subtype of lesions in our sample, which predominantly consisted of ER-positive/HER2-negative cancers. Due to lower blood flow, ER-positive or HER2-negative lesions exhibit lower ADC values than ER-negative or HER2-positive cancers [47, 48]. As ADC is affected not only by tissue cellularity but several pathophysiologic processes including blood flow, membrane permeability and the geometric architecture of the interstitial space [49, 50], it is likely that the lack of association between the PET hypoxia parameters and ADC is a consequence of the combined effect of cellularity, perfusion and microvessel structure on ADC. This assertion is further supported by the weak correlations between DCE-MRI indices and ADC observed in this study. It should be noted, however, that inconsistent correlations between ADC and DCE-MRI parameters have been reported in tumours, including breast cancer [51–53].

We calculated hypoxic fractions based on a specific parameter for hypoxia namely influx rate constant *K*_i_. Despite the higher variability associated with kinetic parameter estimates, our choice was based on two considerations. First, several authors have reported lack of correlation between ^18^F-FMISO uptake ratios and pO_2_ measurements casting doubt on the accuracy of thresholds derived from static PET imaging for hypoxic quantification [54, 55]. Kinetic parameters, including *K*_i_, have provided superior correlations with physiological measures of hypoxia from pO_2_ histography and immunohistochemistry [54, 55]. Second, these thresholds have mostly been defined on measurements from head-and-neck cancers and are not necessarily applicable to other tumour types, including breast cancer.

The main limitations of our study are the small sample size and that the majority of cancers were HR-positive ductal carcinomas. Though our findings cannot be generalised to the full spectrum of histological/molecular subtypes encountered in breast cancer, our study indicates the presence of hypoxia in all histological subtypes studied independent of nuclear grade. While the majority of lesions (56%) examined were found to be non-hypoxic, it should be noted that breast tumours are generally less hypoxic than cancers of the head and neck, cervix or lung and show greater variability in hypoxia among molecular subtypes, with basal-like subtypes being the most hypoxic [56].

Our demonstration of in vivo simultaneous measurement of perfusion and hypoxia is clinically important for three reasons. First, previous reports have indicated that tumours with a high hypoxia-perfusion ratio (i.e. hypoxia due to low perfusion) have a poorer prognosis and suboptimal treatment response [57, 58]. In breast cancer, studies have described differences in the response to perfusion-related hypoxic exposure between molecular subtypes [59, 60], emphasising the need for combined hypoxia-perfusion measurements to provide more accurate prognostic information or tailor treatment. Second, preoperative radiotherapy or radiochemotherapy regimes in early or locally advanced breast cancer have reported beneficial clinical outcomes [61, 62]. Hypoxia and hypoperfusion are known to reduce the effectiveness of radiotherapy and chemotherapy, and the hypoxia-perfusion status of tumours at baseline could allow optimisation of these regimens. Third, tumour hypoxia can occur independently of hypoperfusion as evidenced in the oncology literature [39, 40, 57, 58] and our findings. As such, the data presented here can be viewed as providing further indication of the benefit of non-invasive multimodal assessment of the tumour microenvironment for disease characterisation.

In conclusion, we found a negative relationship between tumour hypoxia, measured by ^18^F-FMISO-PET, and markers of perfusion and vascular function from DCE-MRI, endorsing the hypothesis of perfusion-driven hypoxia in breast cancer. No associations were observed between ^18^F-FMISO-PET parameters and tumour histology or grade, but hypoxic fractions increased with lesion size. The intratumoural heterogeneity observed in hypoxia and perfusion images is consistent with the known complex relationship between perfusion and the hypoxic tumour micromilieu. The combined hypoxia-perfusion status of tumours may need to be considered in determining treatment efficacy or informing therapy selection in breast cancer, which could be achieved using simultaneous multimodality imaging as reported here.

## Electronic supplementary material

ESM 1 (DOCX 1303 kb)

ESM 2 (DOCX 21 kb)

## Abbreviations

%HF: Percentage hypoxic fraction
^18^F-FMISO: ^18^F-fluoromisonidazole
ADC: Apparent diffusion coefficient (mm^2^/s)
*k*_ep_: Contrast efflux rate constant (min^−1^)
*K*_i_: Tracer influx rate constant (mL/cm^3^/min)
*K*^trans^: Contrast influx transfer rate constant (mL/g/min)
SUV: Standardised uptake value (g/mL)
*T*_max_/*M*: Maximum tumour-to-muscle ratio
*T*_max_/*P*: Maximum tumour-to-plasma ratio
*v*_e_: Extravascular-extracellular volume fraction
*v*_p_: Plasma volume fraction
