# Supplementary material for: Hypoxia and perfusion in breast cancer: simultaneous assessment using PET/MR imaging
Source: Eur Radiol. 2020 Jul 28;31(1):333–44. doi: 10.1007/s00330-020-07067-2 (PMC7755870; doi:10.1007/s00330-020-07067-2)

**Supplemental Table 1:** MRI acquisition parameters.

| Acquisition parameters | T_1_ mapping  (VFA) | B_1_^+^ mapping  (Bloch-Siegert) | DCE  (VIBRANT-TRICKS) | DWI |
| --- | --- | --- | --- | --- |
| Sequence | 3D SPGR | 2D SPGR | 3D SPGR | 2D SE-EPI |
| Acquisition plane | Axial | Axial | Axial | Axial |
| FOV diameter (mm) | 350 | 350 | 350 | 360 |
| Image matrix | 256×256 | 128×128 | 512×512 | 140×192 |
| Slice thickness (mm) | 2.8 | 7.0 | 2.8  (interpolated to 1.4) | 4.0 |
| No. of slices | 112 | 22 | 112 | 26 |
| *b*-values (s/mm^2^) | n/a | n/a | n/a | 0, 900 |
| Pixel size (mm) | 1.4×1.4 | 2.7×2.7 | 0.6×0.6 | 2.6×1.9 |
| Fat suppression | No | No | Yes^a^ | Yes^a^ |
| ASSET factor | 2 | n/a | 2.5 | 2 |
| TR (ms) | 4.2 | 24 | 7.1 | 6.0 |
| TE (ms) | 2.1 | 13.7 | 3.8 | 94.9 |
| RF excitation (degrees) | 2, 3, 5, 10, 15 | 20 | 12 | 90 |
| No. of averages | 1 | 1 | 0.5 | 5 |
| Bandwidth (kHz) | 62.5 | 15.6 | 125 | 250 |
| Acquisition time | 33 s  (per flip angle) | 2 m 20 s | 8 m 5 s^b^ | 10 m 48 s |

^a^Spatial-spectral water excitation

^b^Nominal temporal resolution: 10 s per phase

VFA: variable flip angle; VIBRANT-TRICKS: volume image breast assessment–time-resolved imaging of contrast kinetics; 3D SPGR: three-dimensional spoiled gradient recalled echo; 2D SPGR: two-dimensional spoiled gradient recalled echo; 2D SE-EPI: two-dimensional spin echo–echo-planar imaging; FOV: field-of-view; ASSET: array spatial sensitivity encoding technique.

**Supplemental Table 2:** Additional clinical data for the patient population (*n*=29).

| **Characteristic** | ***n* (%)** |
| --- | --- |
| Menopausal status |  |
| Pre-menopausal | 10 (35) |
| Post-menopausal | 19 (65) |
|  |  |
| Clinical management |  |
| Primary surgery | 21 (72) |
| Neo-adjuvant therapy | 8 (28) |
|  |  |
| Lesion presentation |  |
| Unilateral | 26 (90) |
| Unilateral (synchronous) | 1 (3) |
| Bilateral (synchronous) | 2 (7) |
|  |  |
| Lesion laterality^a^ [*n*=32 lesions] |  |
| Right breast | 16 (50) |
| Left breast | 16 (50) |

^a^Lesion numbers include synchronous bilateral carcinomas, which were regarded as independent lesions.

**Supplemental Table 3**: Hotspot *K*^trans^ (mL/g/min) and ^18^F-FMISO-PET parameters with respect to tumour histology. Data are presented as mean ± standard deviation (SD) or median [range] as appropriate.

|  | **Histology** | | | |  |
| --- | --- | --- | --- | --- | --- |
| **Parameter^a^** | **IDC** | **ILC** | **Mixed** | **IMC** | ***p*-value** |
| Lesions (*n*=31) | 20 | 6 | 3 | 2 |  |
| *Hotspot K*^trans^ | 2.50 ± 0.95 | 2.67 ± 0.35 | 1.68 ± 1.05 | 2.24 ± 1.58 | 0.46^a^ |
| *Hotspot K*_i_ (×10^-3^) | -0.03 ± 1.14 | 0.54 ± 0.79 | 0.69 ± 1.59 | 0.39 ± 1.77 | 0.65^a^ |
| *Hotspot* SUV | 0.90 ± 0.26 | 0.91 ± 0.41 | 0.90 ± 0.48 | 1.14 ± 0.75 | 0.68^a^ |
| *Hotspot* T/P | 0.49  [0.33-1.07] | 0.42  [0.23-0.56] | 0.53  [0.27-0.66] | 0.75  [0.66-0.85] | 0.38^b^ |

^a^One-way analysis of variance (ANOVA)

^b^Mood’s median test

*K*^trans^: contrast influx rate (mL/g/min); *K*_i_: ^18^F-FMISO influx rate (mL/cm^3^/min); SUV: standardised uptake value (g/mL); T/P: tumour-to-plasma ratio.

**Supplemental Table 4:** Hotspot *K*^trans^ (mL/g/min) and ^18^F-FMISO-PET parameters in the hotspot area with respect to nuclear grade. Data are presented as median [range] or mean ± standard deviation (SD) as appropriate.

|  |  | **Grade** |  |  |
| --- | --- | --- | --- | --- |
| **Parameter** | **1** | **2** | **3** | ***p*-value** |
| Lesions (*n*=31) | 3 | 15 | 13 |  |
| *Hotspot K*^trans^ | 2.55  [2.30-3.58] | 2.30  [0.53-4.26] | 2.32  [1.75-3.70] | 0.68^a^ |
| *Hotspot K*_i_ (×10^-3^) | -0.43 ± 0.42 | 0.57 ± 0.97 | -0.16 ± 1.31 | 0.15^b^ |
| *Hotspot* SUV | 0.66 ± 0.09 | 0.96 ± 0.25 | 0.94 ± 0.27 | 0.17^b^ |
| *Hotspot* T/P | 0.37  [0.35-0.48] | 0.53  [0.23-0.90] | 0.54  [0.32-1.07] | 0.40^a^ |

^a^Kruskal-Wallis *H*

^b^One-way analysis of variance (ANOVA)

*K*^trans^: contrast influx rate (mL/g/min); *K*_i_: ^18^F-FMISO influx rate (mL/cm^3^/min); SUV: standardised uptake value (g/mL); T/P: tumour-to-plasma ratio.

**Supplemental Figure 1:** ^18^F-FMISO population-based arterial input functions (AIFs) for four representative patients, each scaled by two venous plasma samples.


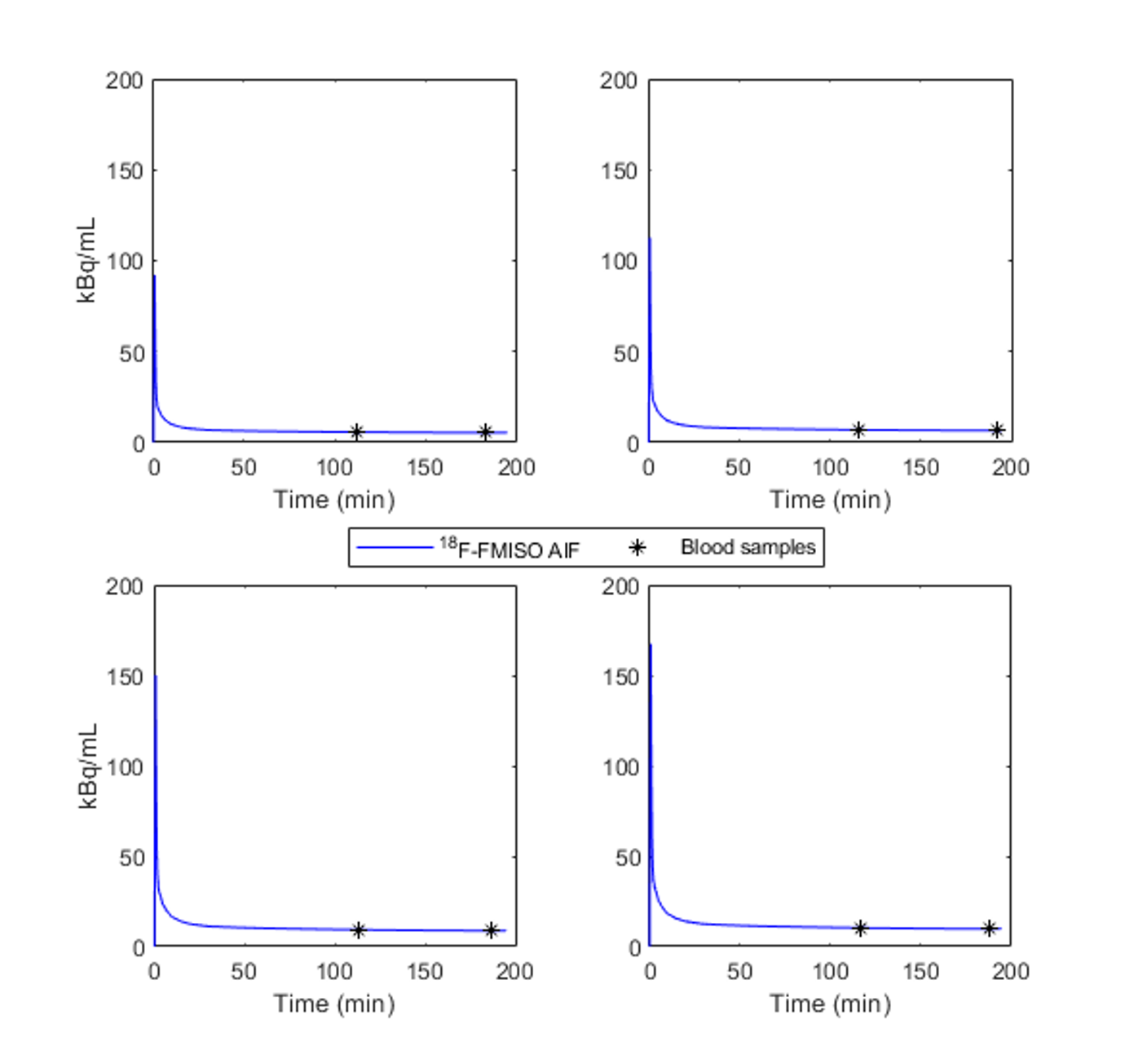


**Supplemental Figure 2:** Axial images of the four representative patients shown in Fig. 2 with: (**a**) invasive ductal carcinoma (IDC); (**b**) invasive lobular carcinoma (ILC); (**c**) invasive mucinous carcinoma (IMC); and (**d**) carcinoma of mixed ductal and lobular type (Mixed). (*Left to right*) DCE-MRI image at peak enhancement, *K*^trans^, *K*_i_, *k*_ep_, *v*_e_ and *v*_p_ maps for the lesion ROI overlaid on the peak-enhancing DCE-MRI image. *K*^trans^: contrast influx transfer rate (mL/g/min); *k*_ep_: contrast efflux transfer rate (min^-1^); *v*_e_: fractional volume of extravascular-extracellular space; *v*_p_: plasma fractional volume; *K*_i_: ^18^F-FMISO influx rate (mL/cm^3^/min).


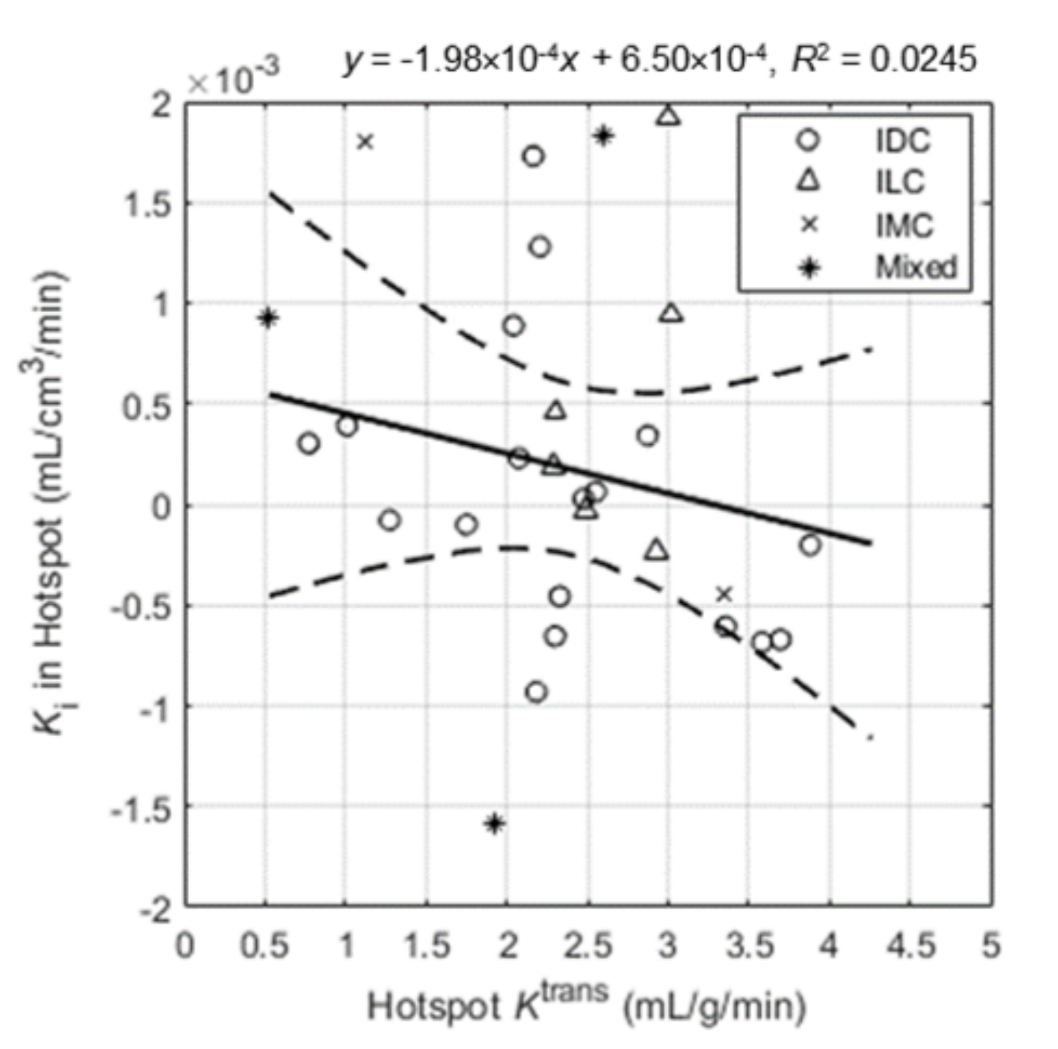


**Supplemental Figure 3**: Scatterplot and regression line of *K*_i_ (mL/cm^3^/min) *vs*. *K*^trans^ (mL/g/min) in the most vascularised area of the tumour (hotspot). Hotspot *K*^trans^ was calculated by averaging pixel values within a 9-pixel square region placed around the area exhibiting the highest *K*^trans^ value on the *K*^trans^ parametric maps [36]. The region encompassing the hotspot *K*^trans^ area was subsequently superimposed on the corresponding co-registered *K*_i_ map to calculate the mean *K*_i_ values within the hotspot area. The Pearson correlation coefficient between *K*_i_ and *K*^trans^ was *r* = -0.16 (*p*=0.40). *K*^trans^: contrast influx rate; *K*_i_: ^18^F-FMISO influx rate.


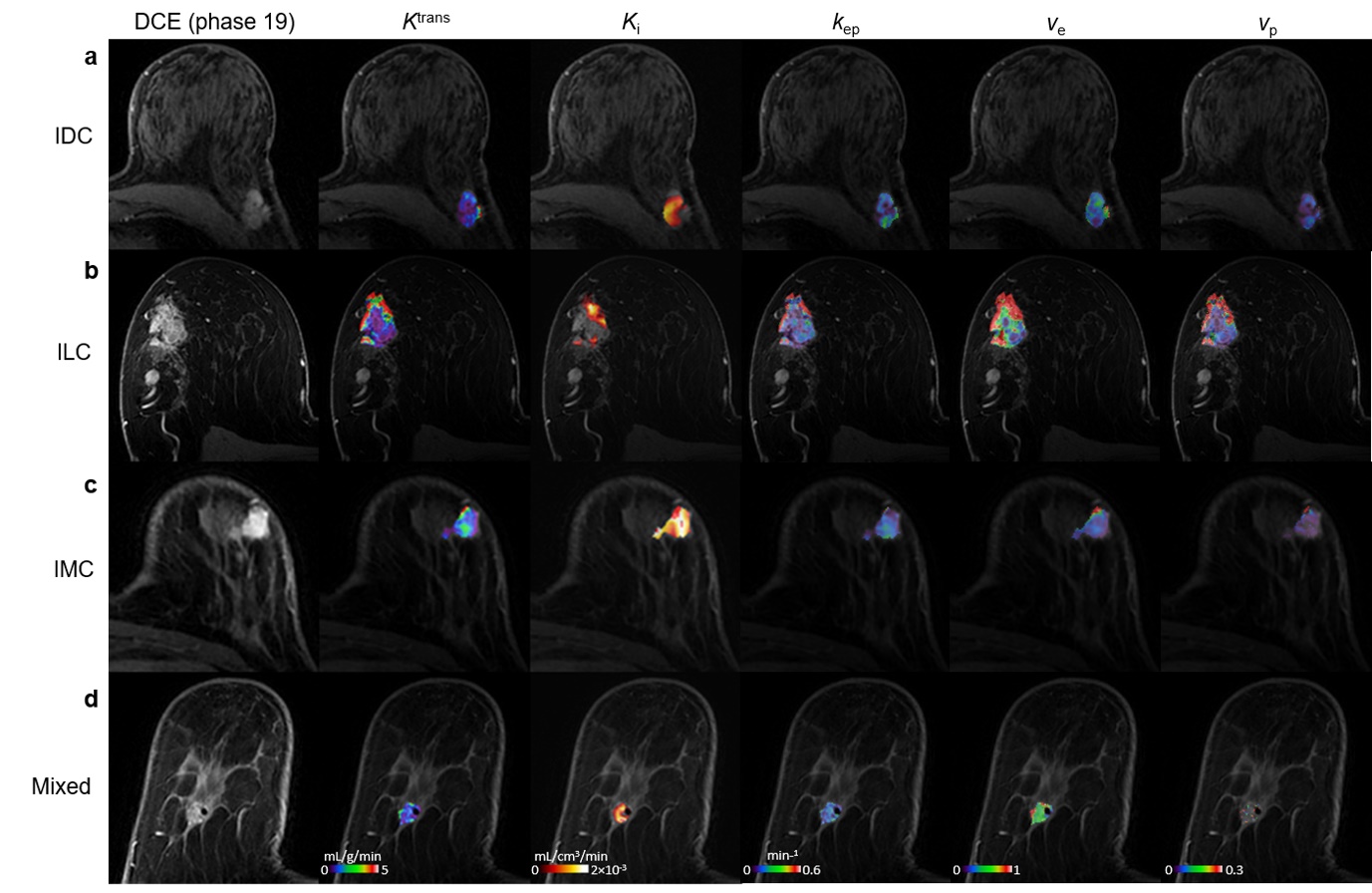

Supplement: Supplementary file 1 — (DOCX 1303 kb) [file 330_2020_7067_MOESM1_ESM.docx]
