# Supplementary material for: Hypoxia and perfusion in breast cancer: simultaneous assessment using PET/MR imaging
Source: Eur Radiol. 2020 Jul 28;31(1):333–44. doi: 10.1007/s00330-020-07067-2 (PMC7755870; doi:10.1007/s00330-020-07067-2)
Supplement: Supplementary file 2 — (DOCX 21 kb) [file 330_2020_7067_MOESM2_ESM.docx]

**Supplementary Material**

**Supplemental Methods**

1. **PET image reconstruction parameters**

A 192×192×89 matrix with 3.12×3.12×2.78-mm voxels used for PET image reconstruction. Corrections for normalisation, dead-time, random events, scatter, attenuation, sensitivity and isotope decay were applied as implemented on the scanner, together with an isotropic 4-mm FWHM Gaussian post reconstruction filter.

1. **Measurement of ^18^F-FMISO radioactivity concentration in blood samples and scaling of the population-based arterial input function (AIF)**

The ^1^^8^F-FMISO population-based arterial input function (AIF) used in this study was generated by averaging measured arterial input functions derived from six healthy volunteers scanned at the *BLINDED* as part of a study in stroke.

For each scan the ^18^F-FMISO population-based AIF was scaled by two venous blood samples (~2 mL each) collected following arteriovenous equilibrium [29], prior to (107 ± 6.4 min) and after the end (186.9 ± 6.5 min) of the PET/MR acquisition. Immediately after collection, each blood sample was aliquoted into a sample tube and centrifuged (6000 rpm; 5 min) to separate plasma, of which ~0.5 mL was apportioned for measuring radioactivity using a Triathler gamma counter (HIDEX). The radioactivity concentration (Bq/mL) in each plasma sample was subsequently calculated accounting for radioisotope decay between the time of measurement and injection. Given the low levels of metabolism and protein binding of ^18^F-FMISO in human plasma, no correction for ^18^F-FMISO plasma metabolites or protein binding was performed [29-31]. To determine the scale factor applied to the ^18^F-FMISO population-based AIF for each patient, the ratio between the measured ^18^F-FMISO radioactivity concentration in each venous plasma sample and the population-derived AIF at the time of blood sampling was calculated and averaged across the two blood samples.

Example AIFs from four representative patients are illustrated in Supplemental Figure 1.

1. **Image Analysis**

*Tumour region-of-interest (ROI) delineation:* Tumour ROIs encompassed the enhancing tumour volume, while visually excluding normal breast parenchyma, necrotic areas and large vessels. To guide region delineation on the DCE images, subtraction images were created in Osirix, version 8.0.2 (Pixmeo SARL), by subtracting pre-contrast images from the peak-enhancing phase of the DCE-MRI series (~2 min from the start of enhancement). For the exclusion of large vessels, maximum-intensity projection (MIP) images were also generated from the subtraction image-set and used as an additional reference for ROI delineation.

For measurement of mean apparent diffusion coefficient (ADC) in lesions, whole tumour regions were demarcated on all axial slices encompassing the tumour on the *b*=900 s/mm^2^ image, using the DCE post-contrast images as guidance, and subsequently propagated on the corresponding ADC map. For ROI definition, care was taken to avoid tumour boundaries, non-enhancing lesion voxels, necrotic and cystic areas [36].

*DCE-MRI*: B_1_^+^-correction maps were generated from the Bloch-Siegert method using in-house software implemented in Matlab R2016b (Mathworks Inc.). T_10_ maps were computed in MIStar, version 3.2.63 (Apollo Medical Imaging) utilising the B_1_^+^-field maps to correct for spatial variations in flip angle. Prior to pharmacokinetic analysis, a cuboid region encompassing the tumour across the DCE-MRI series was motion corrected via a 3D affine model implemented in MIStar, utilising the peak-enhancing phase of the DCE image series as reference for co-registration. Modelling utilised the modified Fritz-Hansen AIF, with all parameters restricted to positive values [32].

*DWI*: ADC maps were calculated using the following equation:

$\text{ADC}=\frac{\text{ln}\left( \frac{S_{0}}{S_{1}} \right)}{\left( b_{1}-b_{0} \right)}$ (1)

where *S*_0_ and *S*_1_ are the signal intensities in images obtained with *b*_0_=0 s/mm^2^ and *b*_1_=900 s/mm^2^, respectively.

*PET*: To reduce the impact of patient motion during acquisition, ^18^F-FMISO dynamic image series were non-rigidly registered to the first frame using the Advanced Normalization Tools (ANTs) package (<http://stnava.github.io/ANTs/>). Registered frames from 150–180 min p.i. were averaged, rigidly registered to the peak-enhancing phase of the DCE-MRI series and subsequently employed for the determination of ^18^F-FMISO uptake (SUV_mean_, SUV_max_, T_max_/P, T_max_/M) in the tumour regions defined on the DCE-MRI. The quality of the registrations was visually inspected by a breast radiologist. For T_max_/M calculations, the mean radioactivity concentration in a bilateral region in the pectoral muscle was used to represent normoxic tissue. In two cases, where lesions were located directly adjacent to pectoral muscle, regions were only placed in the contralateral muscle. Given that increased tracer uptake may represent high tracer delivery to a region rather than trapping under hypoxic conditions, the influx rate of ^18^F-FMISO (*K*_i_) into the trapped tissue compartment was determined as a more specific measure of tumour hypoxia. *K*_i_ maps were produced by Patlak-plot analysis, using in-house software implemented in Matlab R2016b. Image analysis was performed in Analyze 12.0 (AnalyzeDirect Inc.).
